# Informing Equitable Prevention Practices: A Statewide Disaggregated Analysis of Suicide for Ethnoracially Minoritized Adolescents

**DOI:** 10.1007/s11121-024-01654-1

**Published:** 2024-03-02

**Authors:** Sonyia C. Richardson, John A. Williams, Michelle M. Vance, Margaret Phipps-Bennett, Andre P. Stevenson, Rehaana Herbert

**Affiliations:** 1 School of Social Work, University of North Carolina at Charlotte, 9201 University City Blvd, Charlotte, NC 28223 USA; 2 Texas A&M University, College Station, TX USA; 3 North Carolina A&T State University, Greensboro, NC USA; 4 Elizabeth City State University, Elizabeth City, NC USA; 5 University of North Carolina at Greensboro, Greensboro, NC USA

**Keywords:** Ethnoracial, Sexuality orientation, Suicidality, Disaggregated, Intersectionality

## Abstract

The increase in adolescent suicide rates in the United States is a pervasive public health issue, and ethnoracial youth with diverse identities are disproportionately impacted, yet less studied. National planning efforts reinforce state-level approaches to suicide prevention through an equitable lens to prevent adolescent suicide. This study examined disaggregated state-level data over time to determine changes to suicide outcomes based on race/ethnicity, sex, sexual orientation, and the intersection of these identities and determined which sub-groups had higher odds of suicide outcomes. Data from the 1991–2019 Centers for Disease Control and Prevention Youth Risk Behavioral Surveillance System were analyzed for 17,419 ethnoracially minoritized high school adolescents in North Carolina. Descriptive analyses and multinominal logistic regression models were employed. Findings indicated that subgroups within categories of ethnoracial populations, specifically Black female adolescents unsure of their sexual orientation, reported higher rates of suicide attempts. Additionally, Multiracial adolescents reported higher means for suicide consideration and attempts over time. Recommendations for investigating state-level suicide data by focusing on diverse intersecting identities to illuminate areas for potential prevention efforts and support health equity are provided.

As suicide among adolescents has become the second leading cause of death and a national public health issue in the United States (Curtin et al., 2022), the Surgeon General issued an urgent plea to implement the National Strategy for Suicide Prevention, which includes state-level approaches to drive prevention efforts (U.S. Department of Health and Human Services Office of the Surgeon General & National Action Alliance for Suicide Prevention [US DHHS/NAASF], 2021). Acknowledging that suicide outcomes need to be data-driven as they vary by state, region, and demographics (US DHHS/NAASF, 2021), the need to examine suicide outcomes among adolescents with attention to racial and ethnic group differences is reinforced (Bridge et al., 2018; Lindsey et al., 2019). These examinations are necessary to identify differences between groups and to support more targeted prevention efforts for populations disproportionately affected by suicide outcomes (Quinlan et al., 2021).

Researchers have examined trends among ethnoracial (Black, Hispanic, American Indian/Alaska Native, Asian, Pacific Islander, and Multiracial) adolescent groups at a national level (Baiden et al., 2020; Sheftall et al., 2022; Ivey-Stephenson et., 2020); yet very few have analyzed and reported on state-level data. As states maintain an integral role in developing and implementing suicide action plans to guide more comprehensive, systematic, and integrated responses (Quinlan et al., 2021), the analysis of demographic data highlights adolescent risk patterns based on a myriad of identities and their intersections (Baiden et al., 2020; Bath & Njoroge, 2021; Crenshaw, 1991; Sheftall et al., 2022). Further, to support comprehensive suicide prevention strategies, efforts for inclusive and intersectionality-informed research approaches must be realized, allowing for an understanding of the unique needs of diverse identities (Bath & Njoroge, 2021; Bridge, 2018; Opara et al., 2020; Polanco-Roman & Miranda, 2022).

## The Need for Research on Ethnoracially Minoritized Populations

Examining suicide outcomes among ethnoracially minoritized populations reveals varying patterns. The Centers for Disease Control and Prevention  (CDC, 2022) reported a national decrease in the suicide death rate in 2019. However, this was not the case for all ethnoracially minoritized populations; there was only a reported decrease among American Indian/Alaska Native populations during the 2019 reporting year (Ramchand et al., 2021). Thus, researchers argue for the need to examine subgroup data for suicide trends for accurate reporting, suicide prevention efforts, and intentional efforts to mitigate risk among these populations.

For example, using the Centers for Disease Control and Prevention Youth Risk Behavior Survey (YRBS) data, Lindsey et al. (2019) examined suicidal thoughts and behaviors (STB) among US high school adolescents from 1991 to 2017. Findings indicated that Black adolescents, including girls and boys, had significant linear increases over time in suicide attempts. Additionally, in another study, Bray et al. (2021) examined racial differences in statewide suicide mortality trends in Maryland during the COVID-19 pandemic. Findings indicated that suicide death rates doubled for Black residents during one of the periods reviewed, while rates decreased in half for White residents. This study suggested the need for disaggregated suicide data based on race/ethnicity at the state level to identify groups at higher risk.

## Inclusion of Multiple Identities in Suicide Research

Ivey-Stephenson et al. (2020) examined cross-sectional data from the 2019 YRBSS and found that high school adolescents who are lesbian, gay, or bisexual (LGB) had higher rates of reported suicidal ideation and suicide attempts. Additionally, an examination of data from 2009 to 2019 indicated that suicide attempts increased among females and Black adolescents (Ivey-Stephenson et., 2020). The study does not report on the layering of identities (i.e., Black female and Black gay female), which would illustrate the unique experiences of these individuals because of their intersecting identities. While the researchers recommend prevention efforts that target those most at risk (Ivey-Stephensonet., 2020), there is a need to layer the multiple identities to inform tailored prevention efforts.

Xiao et al. (2021) further explored temporal trends in STB among adolescents in the US based on sex and race/ethnicity. Cross-sectional analysis of national data from 183,563 high school adolescents participating in the YRBSS data from 1991 to 2019 revealed differences in trends based on sex and race/ethnicity and STB outcomes, with the largest increases in suicide attempts for high school adolescents being males and Black adolescents. The researchers recommend that suicide prevention efforts are tailored based on sex and race; however, this study did not include identification as LGB (Xiao et al., 2021).

A systematic review of research on STB over 50 years found that existing articles were less likely to report race and ethnicity in addition to LGB status (Cha et al., 2018). The articles were often exclusive of these identities and predominantly included samples of White non-Hispanic adults. This dearth of research demonstrates the gaps in the study of suicide and intersecting identities. The need for research on ethnoracially minoritized populations and the layering of multiple identities is urgently warranted and will create more effective suicide prevention strategies for youth of all identities.

In response to the need to disaggregate data on adolescent demographics to understand suicide outcomes, the current study analyzes STB data in North Carolina, a southeastern state, for high schoolers between 1991 and 2019. We sought to identify suicide trends for ethnoracially minoritized high school adolescents through a multinominal logistic regression model to identify which groups (race/ethnicity, sex, and sexual orientation) are more susceptible to STB to inform state-level suicide prevention efforts. Further, we extend the literature on the suicidal risk of ethnoracial youth by examining the intersection of their identities. We conclude this study with recommendations for how states can better support suicide prevention efforts for diverse groups of ethnoracially minoritized youth through more in-depth analyses and utilization of data.

## Methods

### Data Collection

The data collected for this study is extracted from the CDC YRBSS (2022). The CDC administers the Youth Risk Behavior Survey (YRBS), which monitors health-related behaviors associated with death and disability among adolescents every two years, primarily focusing on public and private school students (CDC, 2022). This data is comprised of high school adolescent responses from 1991 until 2019 in North Carolina. Approximately 459,628 youth attend public high schools (North Carolina Department of Public Instruction, n.d.), and 28,794 attend private high schools (North Carolina Department of Administration, 2022). The racial composition of youth in the state denotes that approximately 25% are Black, 18% are Hispanic/Latino, 4.5% are Multiracial, 3.5% are Asian, 1.2% are American Indian, and 0.1% are Pacific Islanders. Since the study focuses on ethnoracially minoritized adolescents and school enrollment data focuses on these categories, only these students were included. Demographic variables that focused on race, sex, and sexual orientation were included, along with questions that focused on suicide ideation, suicide planning, and suicide attempts. The sample size of this study was *N* = 17,419 ethnoracial high school adolescents from North Carolina participating in the national YRBSS from 1991 to 2019. This study included publicly available data, and a review from the Institutional Review Board was not required. All data were analyzed using STATA 17.0.

### Measures

Students were asked to select their demographic group(s) by race, sex, and sexual orientation. The ethnoracial groups reflected in the sample were American Indian, Asian, Black, Hispanic/Latinx, Pacific Islander, and Multiracial. Regarding sex; students could only select female (dummy coded as 0) or male (dummy coded as 1). Lastly, for sexual orientation, students could select heterosexual = 0, gay or lesbian = 1, bisexual = 2, or not sure/questioning = 3. Furthermore, students were allowed to respond 7 ns about STB*, including (a) During the past 12 months, did you ever seriously consider attempting suicide? (b) During the past 12 months, did you make a plan about how you would attempt suicide? (c) During the past 12 months, how many times did you actually attempt suicide?* For this study, these three questions were labeled suicide ideation, suicide planning, and suicide attempt in the dataset. The variables of suicide ideation and suicide planning were binary coded (0 = No Suicide Ideation or Suicide Plan, 1 = Yes Suicide Ideation or Suicide Plan). For the question about suicide attempts, respondents could indicate that they did not attempt suicide or select the number of times they attempted suicide from 1 to 6. This variable was recoded into a binary outcome with all answers that indicated a suicide attempt grouped into one category (0 = No Suicide Attempts, 1 = Yes Suicide Attempt). No survey weights were used during the data analysis, as particularly given the nuanced manner in which individuals identify themselves; estimating populations could lead to an over or under-representation of some minoritized groups (i.e., Native American girls who do not identify with a sexual orientation) (Gelman, 2007).

### Statistical Analysis

A descriptive analysis was conducted to examine changes in responses over time. Upon conducting the descriptive analysis of the sample, independent regression analyses were conducted using each of the variables of race/ethnicity, sex, and sexual orientation as predictors. These predictor variables were regressed independently with each dependent variable to determine if a relationship existed before attempting a multinominal logistic regression. A multinominal logistic regression was conducted to disaggregate which groups (subgroups) had higher odds of suicidality. This allowed an examination of changes over the years in student responses to the YRBSS measures. According to the models, each of the independent variables had a statistically significant relationship with suicide ideation (*p* < 0.001), suicide planning (*p* < 0.001), and suicide attempt (*p* < 0.05). A multinominal logistic regression analysis was conducted on three independent variables (race/ethnicity, sex, and sexual orientation) and the dependent variables (categorical) of suicide ideation, suicide planning, and suicide attempt. Each of the variables was entered into the model simultaneously. This approach supports the inclusion of multiple categorical variables and allows for localization to predict the probability of suicide outcomes (Watanabe & Kurita, 2008). In conducting a multinominal logistic regression, the assumptions of no collinearity, a linear relationship between the independent and dependent variables, no outliers in the data, and multicollinearity were met. However, due to the sensitive nature of adolescents’ behaviors, individuals will regularly not respond to questions regarding suicide. Numerous respondents skipped over one or multiple questions on suicide. However, consistent with previous literature on suicide (Romanelli et al., 2022), the missing responses were not removed, nor were multiple imputations conducted to make up for missing data as per recommendations from Kolaja et al. (2021). Missing data was not imputed as MCARs were conducted, and the results indicated that responses were missing at random. Odds ratios were listed in the findings.

## Results

### Descriptive Findings

According to Table 1, Black females were the largest group in the sample (*n* = 4709), followed by Black males (*n* = 4143). The smallest racial/ethnic groups represented in the sample were American Indian females (*n* = 305) and males (*n* = 317) and Pacific Islander females (*n* = 91) and males (*n* = 129). In terms of sexual orientation, the majority of adolescents identified themselves as heterosexual (*n* = 6,104), followed by bisexual (*n* = 591), not sure/questioning (*n* = 314), and gay or lesbian (*n* = 283). Finally, in examining sex and sexual orientation, heterosexual males (*n* = 3228) and females (*n* = 3126) were the most selected group in this sample. Demographic data broken down by race, sex, and sexual orientation across the variables of suicide ideation, suicide planning, and suicide attempt are in Table 2. Most notably, over half of the adolescents who identified as gay or lesbian or indicated not sure/questioning reported experiencing suicidal ideation in the past 12 months. Approximately one in five American Indian adolescents who responded to the survey indicated they developed a suicide plan in the last 12 months. Pertaining to the question about attempting suicide in the last 12 months, strikingly, there was a similar number of heterosexual (*n* = 197) and bisexual (*n* = 200) adolescents who indicated yes. Lastly, in this sample, approximately one in ten attempted suicide, one out of six developed a suicide plan, and about one out of five reported experiencing suicide ideations.

**Table 1:** Demographic totals by race/ethnicity, sex, and sexual orientation high school YRBS North Carolina (1991–2019)

Race/ethnicity	Female	Male	Total	Heterosexual	Gay or lesbian	Bisexual	Unsure	Total
American Indian	305	317	622	165	7	15	11	198
Asian	474	549	1023	397	15	20	31	463
Black	4709	4143	8852	2848	148	271	117	3384
Hispanic/Latinx	2038	1916	3954	1901	71	157	105	2234
Pacific Islander	91	129	220	56	10	7	4	77
Multiracial	1040	885	1925	737	32	121	46	936
Total	8657	7939	16596	6104	283	591	314	7292
Sex								
Female				3126	163	500	187	3976
Male				3228	135	112	140	3615
Total				6354	298	612	327	7591

**Table 2:** Demographic totals for variables of suicide ideation, plan, and attempt by race/ethnicity, sex, and sexual orientation high school YRBS North Carolina (1991–2019)

	Suicide ideation			Suicide plan			Suicide attempt		
	Yes	No	Total	Yes	No	Total	Yes	No	Total
American Indian	144	470	614	103	409	512	61	233	294
Asian	165	848	1,013	120	795	915	61	521	582
Black	1301	7400	8701	923	6544	7467	496	4023	4519
Hispanic/Latinx	719	3194	3913	544	3106	3650	268	1654	1922
Pacific Islander	48	164	212	43	149	192	27	91	118
Multiracial	446	1461	1907	328	1313	1641	139	768	907
**Total**	2823	13537	16360	2061	12316	14377	1052	7290	8342
Heterosexual	905	5394	6299	752	5500	6252	197	2001	2198
Gay or lesbian	100	191	291	83	196	279	31	58	89
Bisexual	253	347	600	219	373	592	200	200	261
Unsure	112	210	322	93	228	321	31	96	127
**Total**	1370	6142	7512	1147	6297	7444	459	2355	2675
Female	1957	6897	8854	1379	6493	7872	642	3996	4638
Male	988	7110	8098	795	6286	7081	481	3564	4045
**Total**	2945	14007	16952	2174	12779	14953	1123	7560	8683

**Fig. 1:**
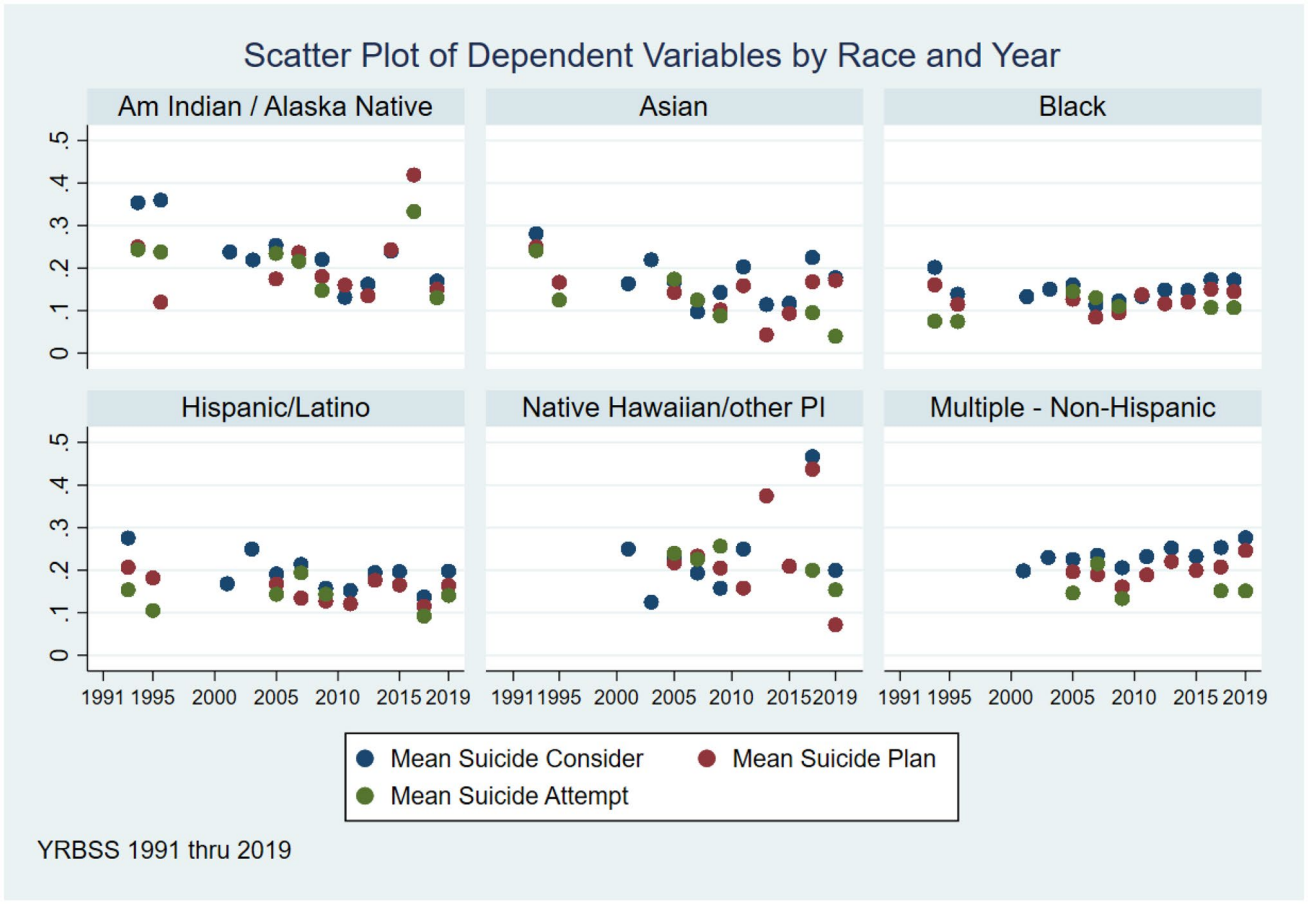
Scatter plot of dependent variables by race/ethnicity and year for high school YRBS North Carolina

Regarding the dependent variables of suicide ideation, suicide plan, and suicide attempt, Fig. 1 shows the means for each variable from 1991 until 2019 by race/ethnicity. Notably, data for the dependent variables were not collected on adolescents in high school who identified as Pacific Islander or Multiracial adolescents until 2000. From 1991 to 2019, the mean response by race/ethnic group for the dependent variables was non-linear. Pertaining to changes over time, there was an overall decrease in the number of youth who responded with “yes” to STB for all racial/ethnic groups except Black and Multiracial adolescents. Multiracial adolescents reported higher means for suicide consideration and attempt over time; yet, according to the findings, there was a slight decrease in the number of Multiracial adolescents who attempted suicide from 2001 (0.18) to 2019 (0.17). Similarly, there were reported decreases in suicide consideration and planning among Black adolescents from 1991 to 2019. However, in 2019, Black adolescents were more likely to attempt suicide (0.18) than in 2001 (0.17). Notably, Pacific Islander adolescents had the highest mean for suicide ideation (0.48) and suicide plan (0.43) during this period, specifically in 2017. During this same year, American Indian adolescents recorded the highest mean for suicide attempts (0.42). Unfortunately, specific groups in certain years (Hispanic/Latinx in 2009; Asian in 2005; Pacific Islander in 2005, 2009, and 2018; Black in 2005, 2007, and 2009; and Multiracial in 2007) reported higher means for suicide attempts than suicide plan. Finally, in the last four years (2015–2019), the means for each dependent variable increased for Hispanic/Latinx, Asian, and Multiracial adolescents in high school.

### Multinominal Logistic Regression

Tables 3, 4, and 5 display the results of the multinominal logistic regression models. Included in this analysis are odd ratios, with the base outcome being no. The reference groups are heterosexual, male, and Hispanic/Latinx adolescents. In examining Table 3, which contains results for suicide ideation as the dependent variable, the model itself was statistically significant. Females had higher odds (OR 2.04; 95% CI 1.56, 2.65) of suicide ideation than males. Black (OR 0.74; 95% CI 0.56, 0.99) adolescents had the lowest odds of all ethnic groups, and multiracial adolescents (OR 1.49; 95% CI 1.04, 2.13) had the highest odds that were statistically significant. Examining the interaction between sex and race, no group had statistically significant odds. However, in examining sexual orientation and suicide ideation, gay and lesbian (OR 2.98; 95% CI 1.30, 6.84), bisexual (OR 6.28; 95% CI 2.85, 14.05), and adolescents selecting the unsure/questioning category (OR 3.04; 95% CI 1.48, 6.23) had higher odds in this category than heterosexual adolescents. Lastly, the interaction between sex, race, and sexual orientation offered no statistically significant results.

**Table 3:** Multinominal logistic regression of sex, race/ethnicity, and sexual orientation on suicide ideation in the last 12 months for the high school YRBS North Carolina

	OR	Std. Err	*z*	(95% CI)	
Yes					
**Sex**					
Female	**2.04**	0.28	5.26	1.56	2.65
**Race**					
American Indian	1.59	0.48	1.53	0.88	2.86
Asian	1.04	0.26	0.16	0.64	1.70
Black	**0.74**	0.11	− 2.05	0.56	0.99
Pacific Islander	0.99	0.61	− 0.02	0.29	3.32
Multiracial	**1.49**	0.27	2.20	1.04	2.13
**Sex and race**					
Female#American Indian	0.77	0.33	− 0.63	0.33	1.77
Female#Asian	0.73	0.24	− 0.94	0.38	1.40
Female#Black	1.16	0.21	0.81	0.81	1.65
Female#Pacific Islander	1.12	0.90	0.14	0.23	5.39
Female#Multiracial	0.82	0.19	− 0.82	0.52	1.31
**Sexual orientation**					
Gay or lesbian	**2.98**	1.26	2.57	1.30	6.84
Bisexual	**6.28**	2.58	4.47	2.81	14.05
Unsure	**3.04**	1.11	3.03	1.48	6.23
**Sex and sexual orientation**					
Female#gay or lesbian	1.08	0.59	0.13	0.37	3.13
Female#bisexual	0.63	0.29	− 1.02	0.26	1.54
Female#unsure	0.71	0.33	− 0.74	0.28	1.77
**Race and sex**					
American Indian#gay or lesbian	3.63	4.81	0.97	0.27	48.78
American Indian#bisexual	0.43	0.57	− 0.64	0.03	5.74
American Indian#unsure	0.00	0.00	− 0.02	0.00	
Asian#gay or lesbian	0.92	0.87	− 0.09	0.14	5.90
Asian#bisexual	1.97	2.02	0.66	0.26	14.69
Asian#unsure	0.39	0.34	− 1.09	0.07	2.13
Black#gay or lesbian	1.46	0.76	0.73	0.53	4.06
Black#bisexual	0.59	0.32	− 0.95	0.20	1.74
Black#unsure	1.03	0.53	0.05	0.37	2.85
Pacific Islander#gay or lesbian	2.91	3.63	0.86	0.25	33.44
*Pacific Islander#bisexual					
*Pacific Islander#unsure					
Multiracial#gay or lesbian	1.29	1.01	0.32	0.28	6.00
Multiracial#bisexual	0.42	0.29	− 1.27	0.11	1.62
Multiracial#unsure	0.63	0.49	− 0.60	0.14	2.88
**Sex, race, and sexual orientation**					
Female#American Indian#gay or lesbian	0.10	0.18	− 1.27	0.00	3.49
Female#American Indian#bisexual	2.86	4.26	0.71	0.15	52.93
*Female#American Indian#unsure					
Female#Asian#gay or lesbian	9.36	14.05	1.49	0.49	177.63
Female#Asian#bisexual	0.40	0.48	− 0.77	0.04	4.17
Female#Asian#unsure	2.40	2.66	0.79	0.28	20.97
Female#Black#gay or lesbian	0.40	0.27	− 1.35	0.11	1.51
Female#Black#bisexual	1.09	0.66	0.14	0.33	3.55
Female#Black#unsure	2.08	1.35	1.13	0.58	7.41
Female#Pacific Islander#gay or lesbian	0.41	0.81	− 0.45	0.01	19.76
Female#Pacific Islander#bisexual	0.00	0.00	− 0.01	0.00	
Female#Pacific Islander#unsure	0.00	0.00	− 0.02	0.00	
Female#Multiracial#gay or lesbian	1.02	0.99	0.02	0.15	6.88
Female#Multiracial#bisexual	2.26	1.72	1.08	0.51	10.02
Female#Multiracial#unsure	2.85	2.59	1.16	0.48	16.92
_cons	0.12	0.01	− 20.11	0.09	0.14

Bold denotes *p* < 0.05. Reference groups: No, Male, Hispanic, Heterosexual

*OR* odds ratio, *95% CI* 95% confidence interval

*N* = 7146; *X*^2^ = 499.59; *R*^2^ = 0.074

^*^Omitted due to collinearity

**Table 4:** Multinominal logistic regression of sex, race/ethnicity, and sexual orientation on suicide plan in the last 12 months for the high school YRBS North Carolina

	OR	Std. Err	*z*	[95% CI]	
Yes					
**Sex**					
Female	**1.59**	0.23	3.21	1.20	2.10
**Race**					
American Indian	1.46	0.47	1.19	0.78	2.73
Asian	0.96	0.25	− 0.16	0.57	1.61
Black	0.82	0.12	− 1.37	0.61	1.09
Pacific Islander	1.92	0.97	1.30	0.72	5.16
Multiracial	**1.73**	0.32	3.03	1.21	2.48
**Sex and race**					
Female#American Indian	0.70	0.33	− 0.76	0.27	1.78
Female#Asian	0.72	0.27	− 0.89	0.35	1.48
Female#Black	1.10	0.21	0.51	0.76	1.60
Female#Pacific Islander	0.58	0.43	− 0.74	0.13	2.50
Female#Multiracial	0.62	0.16	− 1.90	0.38	1.01
**Sexual orientation**					
Gay or lesbian	1.54	0.85	0.78	0.52	4.53
Bisexual	**5.53**	2.41	3.92	2.35	13.00
Unsure	**2.11**	0.86	1.83	0.95	4.68
**Sex and sexual orientation**					
Female#gay or lesbian	2.52	1.66	1.41	0.70	9.13
Female#bisexual	0.74	0.36	− 0.61	0.29	1.91
Female#unsure	1.49	0.75	0.80	0.56	3.98
**Race and sexual orientation**					
*American Indian#gay or lesbian					
American Indian#bisexual	2.28	3.04	0.62	0.17	31.13
*American Indian#unsure					
*Asian#gay or lesbian					
*Asian#bisexual					
Asian#unsure	1.05	0.84	0.07	0.22	5.02
Black#gay or lesbian	1.32	0.88	0.42	0.36	4.88
Black#bisexual	0.45	0.27	− 1.33	0.14	1.45
Black#unsure	1.20	0.67	0.33	0.40	3.60
Pacific Islander#gay or lesbian	1.56	2.23	0.31	0.09	25.77
Pacific Islander#bisexual	1.28	1.28	0.24	0.18	9.11
*Pacific Islander#unsure					
Multiracial#gay or lesbian	0.77	0.74	− 0.27	0.12	5.12
Multiracial#bisexual	0.75	0.51	− 0.43	0.20	2.84
Multiracial#unsure	0.56	0.50	− 0.65	0.10	3.23
**Sex, race, and sexual orientation**					
*Female#American Indian#gay or lesbian					
Female#American Indian#bisexual	0.85	1.28	− 0.11	0.04	16.42
*Female#American Indian#unsure					
*Female#Asian#gay or lesbian					
*Female#Asian#bisexual					
Female#Asian#unsure	0.92	0.97	− 0.08	0.12	7.32
Female#Black#gay or lesbian	0.48	0.39	− 0.91	0.10	2.32
Female#Black#bisexual	1.51	0.98	0.63	0.42	5.40
Female#Black#unsure	0.83	0.57	− 0.27	0.22	3.21
Female#Pacific Islander#gay or lesbian	0.87	1.83	− 0.07	0.01	54.73
*Female#Pacific Islander#bisexual					
*Female#Pacific Islander#unsure					
Female#Muliracial#gay or lesbian	2.01	2.29	0.61	0.22	18.70
Female#Multiracial#bisexual	1.32	1.00	0.37	0.30	5.79
Female#Multiracial#unsure	2.38	2.42	0.85	0.33	17.41
*Male#Pacific Islander#bisexual					
_cons	0.11	0.01	− 20.13	0.09	0.13

Bold denotes *p* < 0.05. Reference groups: No, Male, Hispanic, Heterosexual

*OR* odds ratio, *95% CI* 95% confidence interval *N* = 7,086; *X*^2^ = 401.50; *R*^2^ = .067

^*^Omitted due to collinearity

**Table 5:** Multinominal logistic regression of sex, race/ethnicity, and sexual orientation on suicide attempt in the last 12 months for the high school YRBS North Carolina

	OR	Std. Err	z	[95% CI]	
Yes					
**Sex**					
Female	1.30	0.33	1.04	0.79	2.16
**Race**					
American Indian	1.73	0.99	0.95	0.56	5.30
Asian	0.53	0.29	− 1.17	0.18	1.54
Black	0.72	0.20	− 1.15	0.41	1.26
*Pacific Islander					
Multiracial	1.11	0.41	0.28	0.54	2.30
**Sex and race**					
Female#American Indian	1.60	1.18	0.63	0.38	6.80
Female#Asian	0.58	0.44	− 0.72	0.13	2.59
Female#Black	1.14	0.42	0.35	0.55	2.34
*Female#Pacific Islander					
Female#Multiracial	0.89	0.43	− 0.24	0.34	2.31
**Sexual orientation**					
Gay or lesbian	3.60	3.02	1.53	0.69	18.64
Bisexual	1.80	1.97	0.54	0.21	15.46
Unsure	**8.63**	4.43	4.21	3.16	23.58
**Sex and sexual orientation**					
Female#gay or lesbian	2.30	2.37	0.81	0.30	17.40
Female#bisexual	0.72	0.85	− 0.28	0.07	7.20
Female#unsure	**0.13**	0.10	− 2.67	0.03	0.58
**Race and sexual orientation**					
*American Indian#gay or lesbian					
*American Indian#bisexual					
American Indian#unsure	1.31	1.86	0.19	0.08	21.05
Asian#gay or lesbian	2.85	4.47	0.67	0.13	61.88
Asian#bisexual	3.80	6.36	0.80	0.14	100.91
*Asian#unsure					
Black#gay or lesbian	1.19	1.23	0.17	0.16	9.01
Black#bisexual	2.77	3.61	0.78	0.22	35.47
Black#unsure	0.22	0.20	− 1.64	0.03	1.34
Pacific Islander#gay or lesbian	0.28	630.89	0.00	0.00	
Pacific Islander#bisexual	3.08	5.16	0.67	0.12	81.77
*Pacific Islander#unsure					
*Multiracial#gay or lesbian					
Multiracial#bisexual	1.80	2.53	0.42	0.12	28.25
Multiracial#unsure	0.56	0.59	− 0.54	0.07	4.45
**Sex, race, and sexual orientation**					
*Female#American Indian#gay or lesbian					
*Female#American Indian#bisexual					
*Female#American Indian#unsure					
Female#Asian#gay or lesbian	0.58	1.24	− 0.26	0.01	38.45
Female#Asian#bisexual	2.07	3.98	0.38	0.05	89.45
*Female#Asian#unsure					
Female#Black#gay or lesbian	0.30	0.39	− 0.92	0.02	3.93
Female#Black#bisexual	0.88	1.23	− 0.09	0.06	13.59
Female#Black#unsure	**20.38**	24.07	2.55	2.01	206.38
*Female#Pacific Islander#gay or lesbian					
*Female#Pacific Islander#bisexual					
*Female#Pacific Islander#unsure					
*Female#Multiracial#gay or lesbian					
Female#Multiracial#bisexual	1.96	2.98	0.44	0.10	38.48
Female#Multiracial#unsure	1.63	2.64	0.30	0.07	38.87
_cons	0.09	0.02	− 12.26	0.06	0.14
**No**		Base outcome			

Bold denotes *p* < 0.05. Reference groups: No, Male, Hispanic, Heterosexual

*OR* odds ratio, *95% CI* 95% confidence interval

*N* = 2537; *X*^2^ = 141.50; *R*^2^ = 0.078

^*^Omitted due to collinearity

Using suicide planning as the dependent variable, the model was statistically significant overall. Again, females were found to have higher odds of planning suicide than males (OR 2.49; 95% CI 1.20, 2.10). Only Multiracial adolescents were found to have higher odds than Hispanic/Latinx adolescents (OR 1.73; 95% CI 1.21, 2.48). Combining the categories of sex and race, no statistically significant results were found. Adolescents who identified as bisexual (OR 5.53; 95% CI 2.35, 13.00) or unsure/questioning (OR 2.11; 95% CI 0.95, 4.68) were at higher risk of developing a suicide plan than heterosexual adolescents.

Finally, Table 5 presents the model where suicide attempt was the dependent variable. The model itself was statistically significant. The variables of not sure/questioning (OR 8.63; 95% CI 3.16, 23.58) and Black female adolescents who selected unsure/questioning as their sexual orientation (OR 20.38; 95% CI 2.01, 206.38) had higher odds of attempting suicide, while females who were not sure/questioning (OR 0.13; 95% CI 0.03, 0.58) were less likely to report attempting suicide.

## Discussion

### Understanding Statewide Suicide Trends for Ethnoracially Minoritized High Schoolers

Prior research has primarily examined national and state-wide STB data from an aggregated level with limited attention to the layering of multiple identities (Baiden et al., 2020; Crenshaw, 1991; Ramchand et al., 2021). The first aim of this study was to understand statewide suicide trends for ethnoracially minoritized high school adolescents from a state-level perspective by investigating North Carolina data. Our findings indicate that Black adolescents are the ethnoracial minoritized group with the largest number of adolescents participating in the YRBS in North Carolina since its conception. Cumulatively, over time, Black adolescents have had the highest number of reports of suicide ideations, plans, and attempts. On the other hand, American Indian populations have the highest percentage of adolescents reporting suicide ideation (30.6%), and Pacific Islander has the highest percentage of adolescents reporting suicide planning (28.8%) and suicide attempts (29.6%). These findings illustrate that disaggregation of ethnoracial subgroups and suicide outcomes data at the state level needs to include total numbers and percentages to portray an adequate representation.

Further, the American Indian population is vastly underrepresented in the NC YRBS data. However, there has been a 73% increase in the population within the last ten years in the state (North Carolina Office of State Budget and Management (NCOSBM), 2021). The American Indian population is the fastest-growing racialized group in 37 counties in NC, and 19 counties have seen at least a 200% increase (NCOSBM, 2021). Their lack of representation in participating in this study is an area of concern. As the YRBS uses population-based sampling strategies and the overall representation of this population in the state is proportionately lower, it would be helpful to consider other ways to capture input from this population, including qualitative research and other surveys.

The lack of representation in a state should not discount the percentage of the population impacted by reported suicide outcomes. We argue that all data points should be included for ethnoracial groups for sensitive suicide data to account for the stigma associated with suicide and to ensure each adolescent is seen and counted.

Findings from this study indicate that STB is a prevalent and pervasive issue among racialized and minoritized adolescents, which corresponds to numerous studies (Baiden et al., 2020; Bridge et al., 2018; Quinlan et al., 2021; Sheftall et al., 2022). Though the results indicate that state-level rates of STB have been trending towards zero from 1991 to 2019, there has been an increase in reported STB since 2015. Additionally, there has not been a decrease in STB for adolescents who identified as Multiracial, highlighting these adolescents’ unique and challenging experiences. Although a vastly understudied population, individuals from multiple races are known to experience increased rates of STB, suggested as being attributed to increased levels of depressive symptoms, conflicts about identity, and high rates of rejection (Benton, 2022; Cheref et al., 2015).

The scatterplot of STB patterns over the calendar years of 1991–2019 demonstrated that rates peak for several ethnoracial groups during different spans across our data. For example, for suicide ideation, rates peaked for four subgroups (American Indian/Alaska Native, Asian, Black, and Hispanic/Latino) between 1990 and 1995. For suicide planning, rates peaked during 2015–2019 for three groups (American Indian/Alaska Native, Native Hawaiian/Other PI, and Multiracial/Multiple-Non-Hispanic). Finally, for suicide attempts, rates were higher for all groups during 2005–2010. It would be helpful to understand state-level contextual factors that may have influenced suicide rates. An awareness of these factors may provide more insight into suicide prevention activities. For example, Richardson et al. (2022) examined differences in STB between heterosexual and sexually minoritized adolescents in a southeastern state with exclusionary policies. Further investigation is needed to understand state-level contextual factors influencing suicide rates and strategies to address them.

Xiao et al. (2021) examined suicide trends over time for adolescents by sex and race/ethnicity, but did not include LGB as a variable. With the inclusion of the LGB variable and attention to the multiple race identities, differences were noted based on these intersecting identities. Thus, we recommend that suicide prevention efforts are tailored based on sex, race/ethnicity, and LGB status, including not sure/questioning, and that all groups are prioritized.

### Assessing Suicide Trends Based on Sex and Sexual Orientation

The second aim of this study was to assess variations in these trends based on sex and sexual orientation. Ethnoracially minoritized adolescents who self-identified as female had a significantly higher likelihood of reporting suicide ideation and planning than their male peers. For example, Black females unsure of their sexual orientation had a 20.38% higher likelihood of reporting suicide attempts in North Carolina. This finding corresponds with extant literature on suicide outcomes regarding ethnicity and sex (Alvarez et al., 2022; Baiden et al., 2020; Sheftall et al., 2022). Furthermore, as we inserted sexual orientation into our model, the findings supported previous research: ethnoracially minoritized youth who endorsed being unsure about their sexual orientation and youth identifying as LGB or not sure/questioning had higher rates of ideation and planning (Baiden et al., 2020; Ream, 2019). These intersecting identities play a crucial role in understanding unique experiences with suicide risk. As suggested by Baiden and colleagues (2020), “the intersection of race/ethnicity and sexual minority might further exacerbate distal and proximal stressors on LGBQ adolescents of color” (p. 19), requiring further examination. Targeted support for these groups is recommended.

Our findings extend current recommendations that suicide prevention and implementation efforts for ethnoracially minoritized youth must be approached from the ground up (Sheftall & Miller, 2021). Based on our study, a ground-up approach would begin with an analysis of demographics for existing suicide data to understand suicide risk for ethnoracial populations and subgroups. Several studies across interdisciplinary fields (e.g., education, criminal justice, mental health, etc.) advocate for the disaggregation of data to better pinpoint prevention strategies (Richardson et al., 2023; Morey et al., 2022), and this approach should be applied to suicide data (Quinlan et al., 2021). Additionally, it is important that all stakeholders with a modicum of interactions with youth understand the nuanced identity of a youth experiencing suicide ideation, planning, or attempting to die by suicide. Further, disaggregation of the demographic data allows states to create equitable suicide action plans that are attentive to risk profiles for the entire youth population in the state. Most importantly, this data can inform the prioritization of inclusive voices in the planning process, thereby creating more effective and meaningful suicide interventions (Walsh et al., 2022).

Current methods for examining aggregated data at the state level may not provide a complete understanding of subgroups of populations at the highest risk of suicide. As states can adapt questions on the YRBS, North Carolina and other states should consider adding additional identity-related questions to account for diverse adolescents. Further, intersecting identities experienced by many adolescents in the state may be disregarded as they are currently grouped into more dominant categories. Based on our analysis, we encourage states to add additional questions and then disaggregate their adolescent suicide data regularly (at a minimum yearly). These steps are necessary for planning culturally responsive suicide prevention efforts, which can help to reduce disparities and suicide risk among ethnoracially minoritized adolescents (Vance et al., 2022). For example, based on our research in this state, Black girls who indicated they were unsure of their sexual orientation have a higher likelihood of reported STB. The not/sure questioning identity, which is also more prone to experience increasing rates of STB (Richardson et al., 2022), and its intersection with the Black girl identity require additional investigation.

## Limitations

When interpreting findings from any sample, limitations must be considered. This data is over time and includes different interactions between categorical variables, and the results do not imply causality. First, the missing data is not at random, as suicide is still considered a sensitive/taboo subject to discuss. This would preclude adolescents from answering these questions. Additionally, sex is listed as a binary variable, which is exclusive of adolescents of other identities. The YRBSS asks mainly about sex, but there are additional sexual orientation and gender identity modules that each state can opt into to ask about gender. North Carolina does not include this module, and this limits the findings. Furthermore, this data encompasses all of North Carolina without attention to spatial density, climates, and differences in how ethnoracial and minoritized adolescents are treated in urban, suburban, and rural schools/districts. Having this distinction could offer clues into why adolescents in specific locales are more likely to consider, plan, or attempt suicide based on the abundance or lack of resources (mental, physical, and emotional) that are located either in the school or in the community. Furthermore, adolescents’ self-reported responses could be biased due to their misinterpretation of suicide ideation, suicide plans, and suicide attempts. Other possibilities include mistrust of the survey, recall bias, and stigma. Additionally, this sample does not reflect multiple responses from respondents over time. Future research should attempt to monitor adolescents’ suicidal behaviors over time to pinpoint factors (cultural, social, and emotional) that influence their responses and seek to understand protective factors for intersecting identities that may exist.

## Conclusion

Suicide data at the national level is primarily examined using aggregated analyses and presents a limited understanding of suicide risk based on a myriad of identities. Over 20 years, differences in STB for ethnoracially minoritized adolescents have been noted. Suicide prevention efforts developed and implemented at the state level without immediate attention to disaggregated demographics overlook the impact of intersecting identities. The findings of this study enforce the need for state-level disaggregated analyses to identify disparities in suicide rates, specifically for ethnoracial adolescents with layering identities. These analyses are critical for developing and implementing suicide prevention programs that meet the specific and unique needs of ethnoracially minoritized youth.

## Data Availability

All data analyzed for this study are available for download at https://www.cdc.gov/healthyyouth/data/yrbs/data.htm.
